# Raman spectroscopy data related to the laser induced reduction of graphene oxide

**DOI:** 10.1016/j.dib.2021.107306

**Published:** 2021-08-18

**Authors:** Vittorio Scardaci, Giuseppe Compagnini

**Affiliations:** Dipartimento di Scienze Chimiche, Viale Andrea Doria 6, 95125 Catania, Italy

**Keywords:** Laser synthesis, Graphene oxide, Raman spectroscopy, Controlled atmosphere

## Abstract

This data paper reports data obtained from the fitting of Raman spectra obtained during a laser reduction process for graphene oxide under different processing and material conditions. In particular, we show examples of fitting curves of three different representative reduced graphene oxide spectra, as well as fitting curves for a graphene oxide spectrum. Moreover, we show and compare cumulative distributions of the I_D_/I_G_ values (intensity ratio of peaks D and G) obtained from spectra acquired from different samples. Fittings and distributions were obtained using the OriginPro 8.5 software package. Such data may be the starting point of further experiments on the laser induced reduction of graphene oxide.

## Specifications Table

**Table:** 

Subject	Material Characterization
Specific subject area	Raman spectroscopy of Carbon materials
Type of data	Graph
How data were acquired	Laser scribing: Qiilu DK-BL machine. Laser wavelength: 405 nm. Power: 1.5W Raman: WITec Alpha 300 RS spectrometer. Excitation: 532 nm Spectra fitting: OriginPro 8.5 (https://www.originlab.com/viewer/)
Data format	Raw Analyzed
Parameters for data collection	Each Raman spectrum was obtained acquiring a spectrum with the grating (1800/mm) centred at 1600 cm^−1^ and a spectrum with the grating centred at 2600 cm^−1^. Each spectrum was obtained integrating 10 × 10 second acquisitions
Description of data collection	Samples were prepared in two different atmospheres (Argon and Argon containing 5% H_2_). We used three different scan speeds (1.2, 2.7, 5.9 mm/s), two levels of material coverage (400 and 850 µg/cm^2^) and a single or double laser pass. For each sample, 20 spectra were collected at random locations, the peaks were fitted using Lorentzian curves and the fitting parameters were statistically analysed.
Data source location	Institution: Università degli Studi di Catania City/Town/Region: Catania Country: Italy Latitude and longitude (and GPS coordinates, if possible) for collected samples/data: 37.5269491536957, 15.077758982344017
Data accessibility	Repository name: Mendeley Data Data identification number: http://dx.doi.org/10.17632/9smmfc9vb8.1[1] Link: http://dx.doi.org/10.17632/9smmfc9vb8.1
Related research article	V. Scardaci, G. Compagnini, Raman Spectroscopy Investigation of Graphene Oxide Reduction by Laser Scribing, C 7, (2021) 48 [2]

## Value of the Data

• Data presented provide an insight into the efficiency of graphene oxide laser induced reduction under different conditions.
• Data presented should be of particular interest for researchers in the fields of laser modification of materials and laser synthesis of graphene.
• Data presented may be a starting point for an investigation of laser reduction of graphene oxide under a much broader set of conditions.

## 1 Data Description

**Fig. 1:**
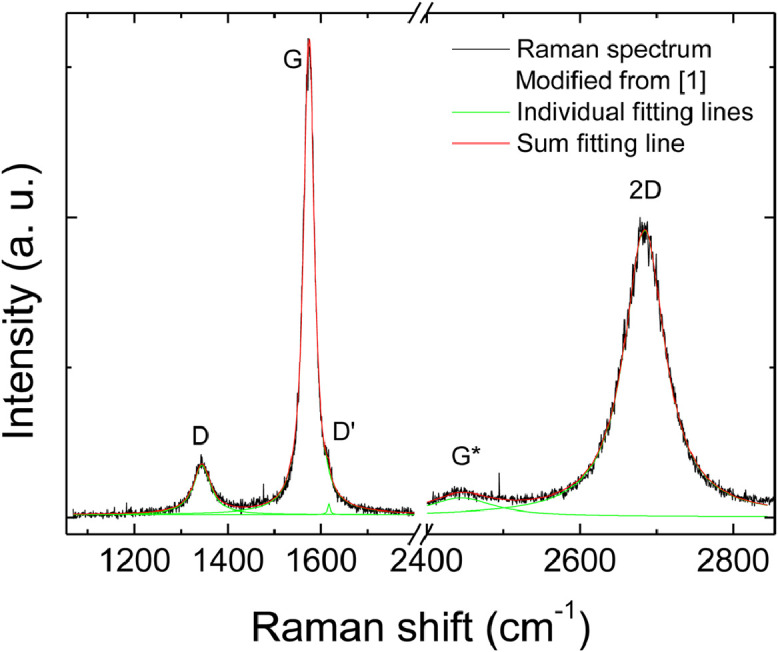
Example of fitting of a Raman spectrum from a high reduction (low I_D_/I_G_) location.

Fig. 1 provides an example of fitting of a Raman spectrum of RGO with a very low I_D_/I_G_ (∼0.2). It can be observed that the low wavenumber region can be fitted by three lorentzian peaks, attributed as in the figure labels, and the high wavenumber region can be fitted by two peaks, attributed as in the figure. The sum fitting line perfectly fits the spectrum.

**Fig. 2:**
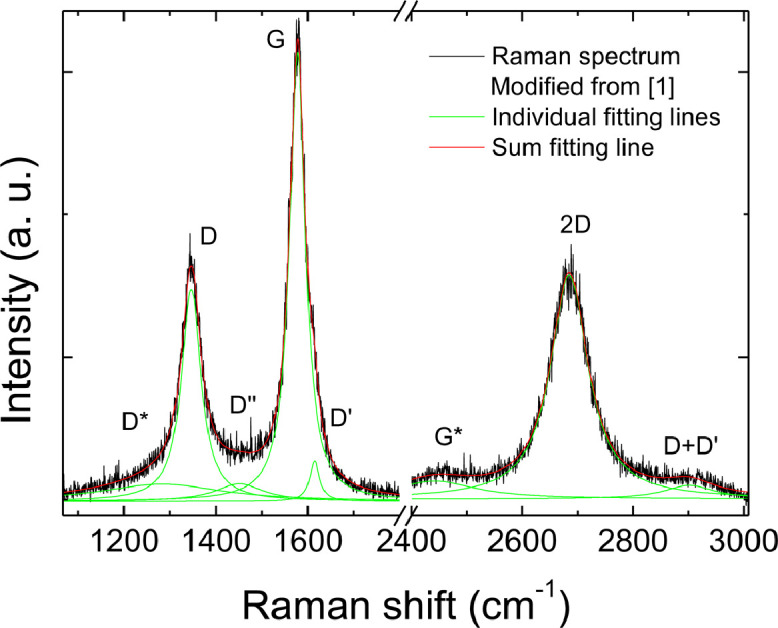
Example of fitting of a Raman spectrum from a medium reduction (medium I_D_/I_G_) location.

**Fig. 3:**
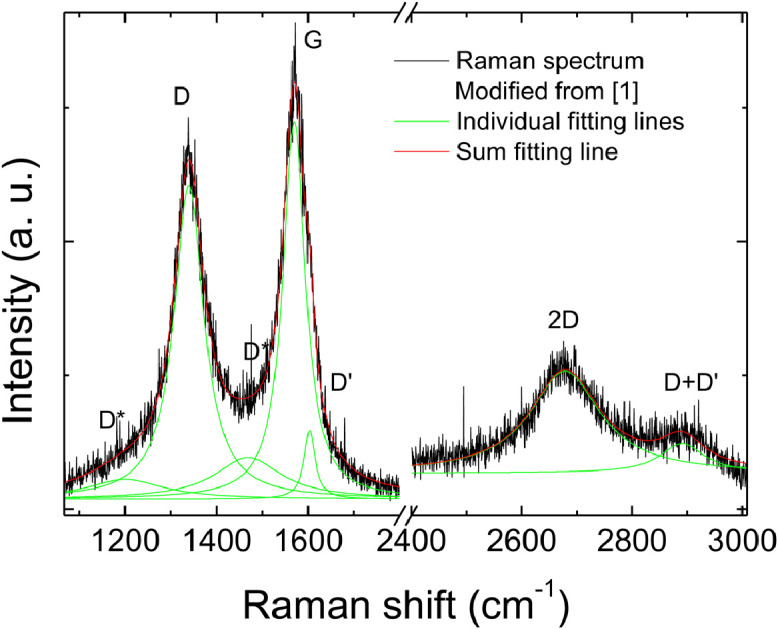
Example of fitting of a Raman spectrum from a low reduction (high I_D_/I_G_) location.

**Fig. 4:**
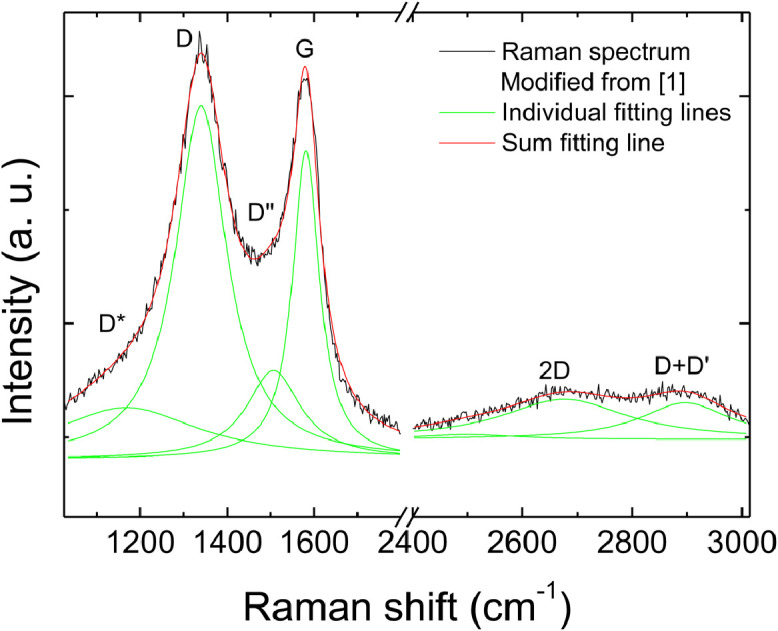
Example of fitting of a Raman spectrum from our GO starting material.

Fig. 2 provides an example of fitting of a Raman spectrum of RGO with a medium I_D_/I_G_ (∼0.5). It can be observed that the low wavenumber region can be fitted by five lorentzian peaks, attributed as in the figure labels, and the high wavenumber region can be fitted by three peaks, attributed as in the figure. The sum fitting line perfectly fits the spectrum.

**Fig. 5:**
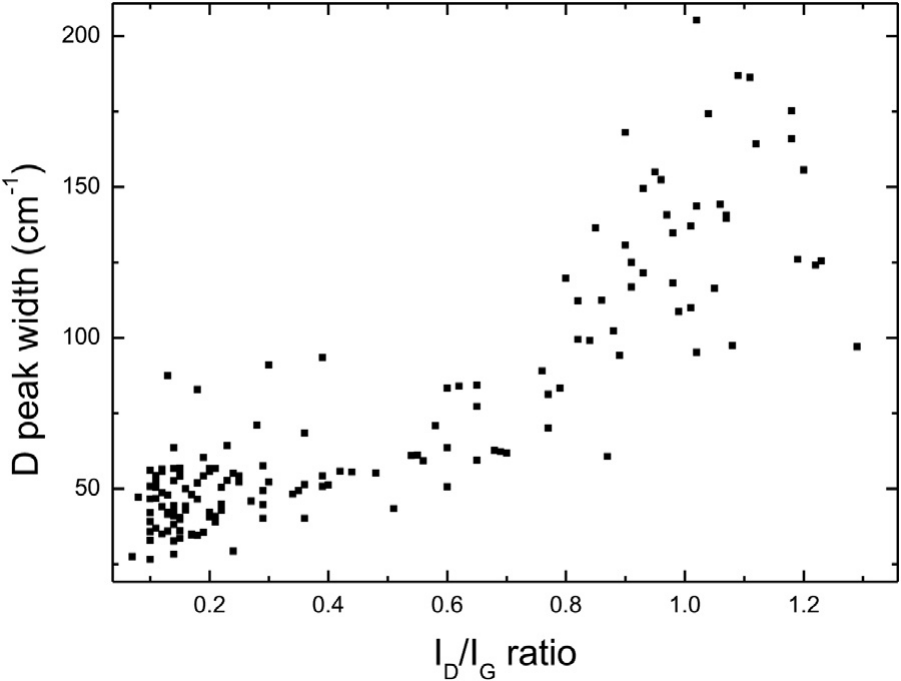
Plot of D peak width versus I_D_/I_G_ ratio for all our data.

**Fig. 6:**
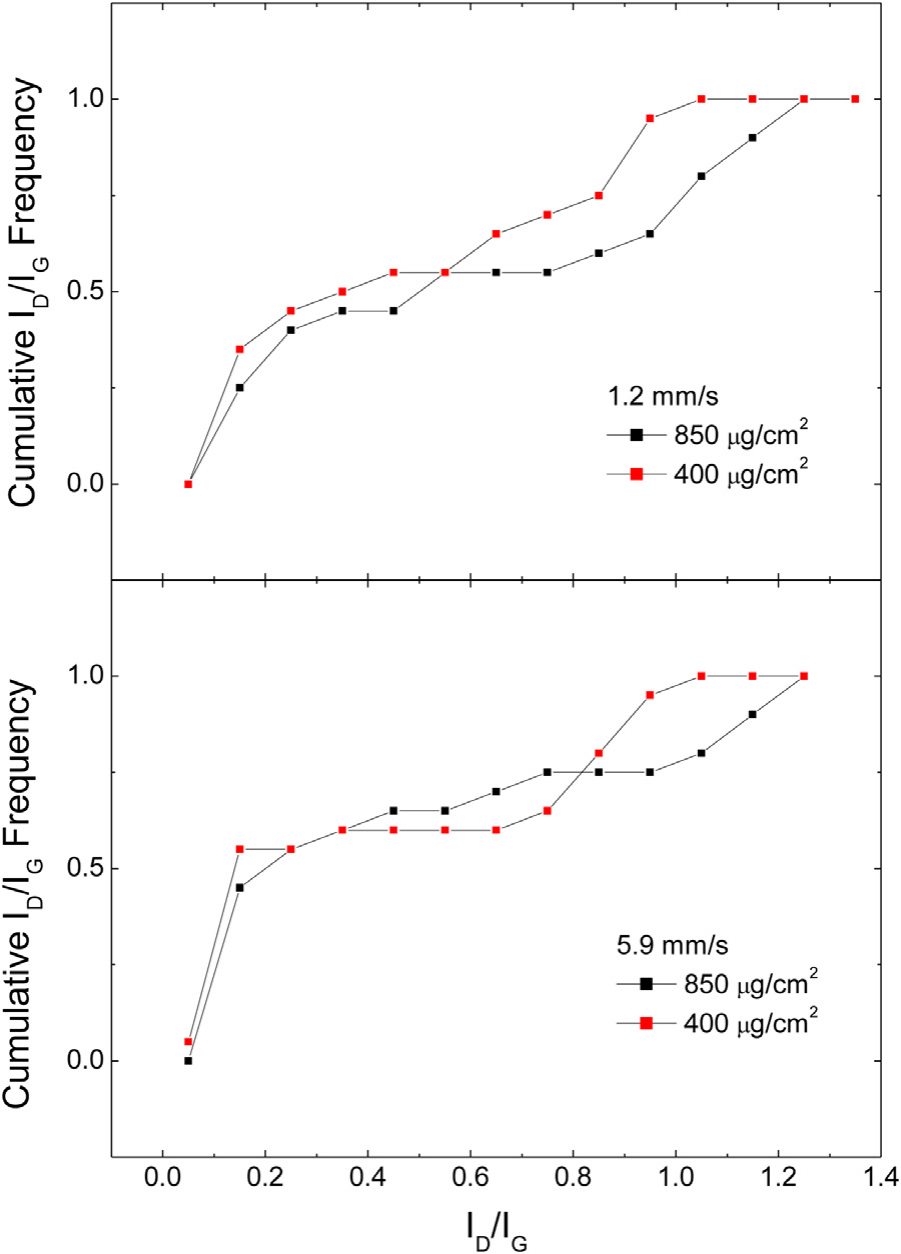
Cumulative I_D_/I_G_ frequencies for samples produced in Argon comparing samples with material coverage 400 and 850 µg/cm^2^.

Fig. 3 provides an example of fitting of a Raman spectrum of RGO with a medium I_D_/I_G_ (∼0.8). It can be observed that the low wavenumber region can be fitted by five lorentzian peaks, attributed as in the figure labels, and the high wavenumber region can be fitted by two peaks, attributed as in the figure. The sum fitting line perfectly fits the spectrum.

Fig. 4 provides an example of fitting of a Raman spectrum of GO. It can be observed that the low wavenumber region can be fitted by four lorentzian peaks, attributed as in the figure labels, and the high wavenumber region can be fitted by two peaks, attributed as in the figure. The sum fitting line perfectly fits the spectrum.

Fig. 5 shows a plot of the full width at half maximum for the D peak, obtained by fitting all our spectra, against the relative I_D_/I_G_ ratio. The trend shows a monotone increase, taking into account the scattering of the data.

Fig. 6 shows the cumulative distribution of the I_D_/I_G_ data, obtained by fittings like those in Fig. 1, Fig. 2, Fig. 3, for samples obtained in Argon at different material coverage and different scan speeds

**Fig. 7:**
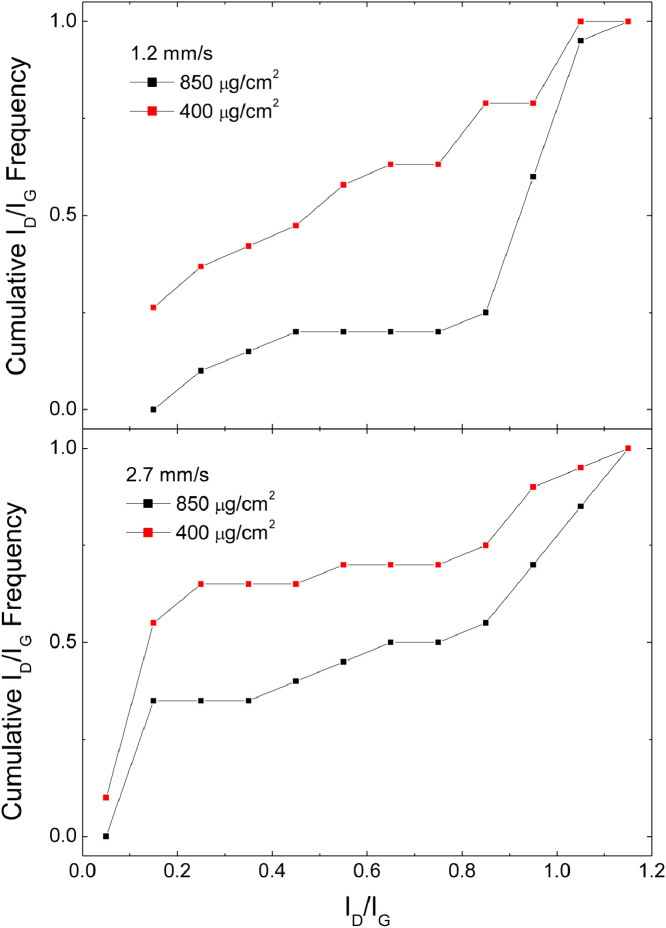
Cumulative I_D_/I_G_ frequencies for samples produced in Argon/H_2_ comparing samples with material coverage 400 and 850 µg/cm^2^.

Fig. 7 shows the cumulative distribution of the I_D_/I_G_ data, obtained by fittings like those in Fig. 1, Fig. 2, Fig. 3, for samples obtained in Ar/H_2_ at different material coverage and different scan speeds

**Fig. 8:**
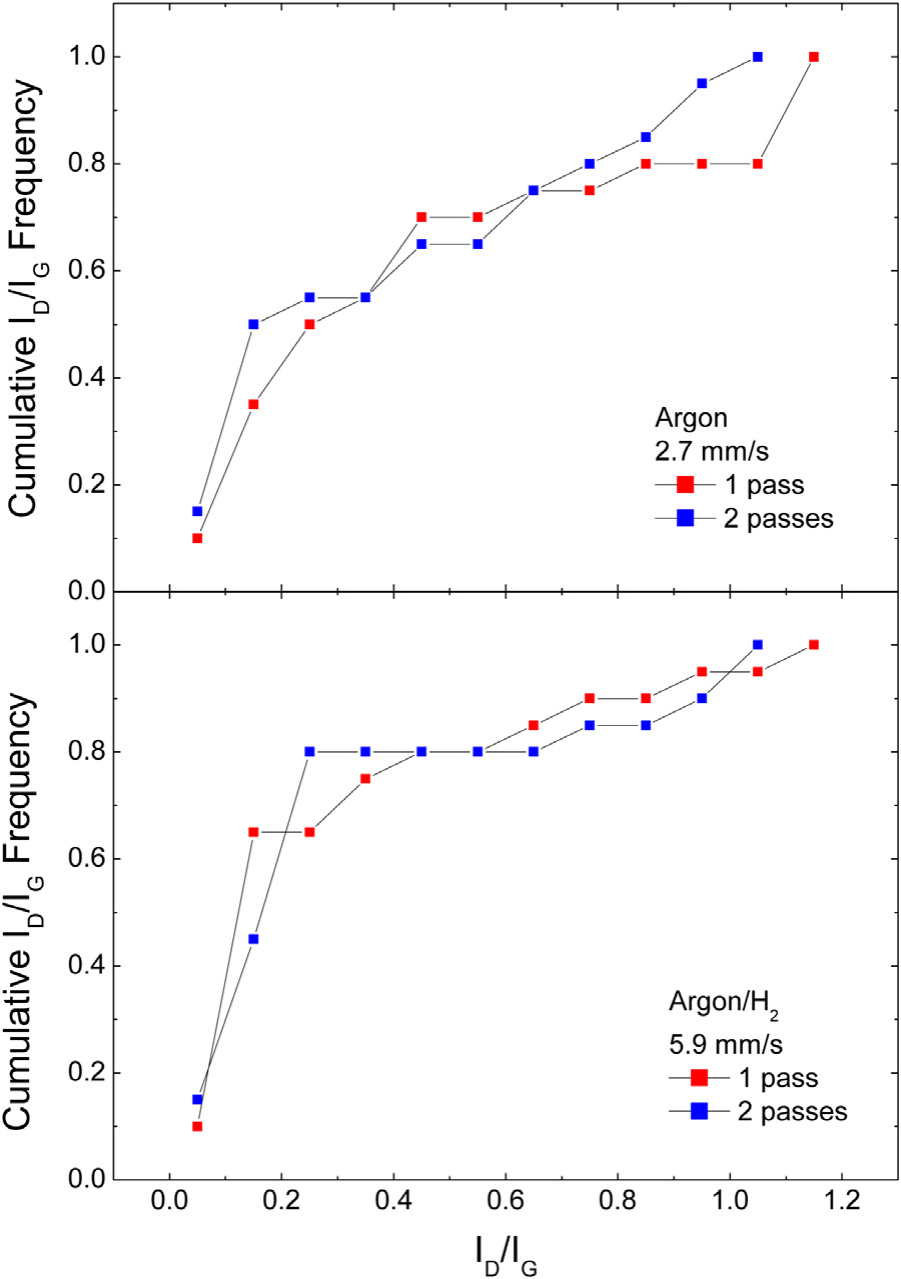
Cumulative I_D_/I_G_ frequencies for samples with 400 µg/cm^2^ material coverage under single and double laser pass.

Fig. 8 shows the cumulative distribution of the I_D_/I_G_ data, obtained by fittings like those in Fig. 1, Fig. 2, Fig. 3, for samples obtained in Argon and Ar/H_2_, at 400 µg/cm2 material coverage, for a single and a double laser scribing pass

Files uploaded on the data repository:

Raw data file: spettri_mend.opj
This file contains raw data for Fig. 1, Fig. 2, Fig. 3, Fig. 4
Raw data file: fit new_mend.opj
This file contains raw data for Fig. 5
Raw data file: Spessore.opj
This file contains raw data for Figs. 6–7
Raw data file: dati 2 pass.opj
This file contains raw data for Fig. 8
Folder: Raman spectra

Origin files containing the raw spectra and the relative fittings. Names are assigned as atmosphere_scan speed_material coverage (e.g. Ar_266_400). Where the number of passes was investigated, files are named as atmosphere_scan speed_material coverage_passes (e.g. Ar_266_400_2P)

## 2 Experimental Design, Materials and Methods

The starting graphene oxide material was purchased from Graphenea as a 0.4%wt water solution and drop-casted in fixed volumes (400 and 850 µl) onto 2 × 2 cm^2^ polyethylene terephthalate (PET) substrates, after a 30-minute bath ultrasonication.

After drying under ambient conditions for two days, substrate were subjected to laser scribing under controlled atmosphere using a disposable glove box (AthmosBag) and a flow of Argon or a mixture of Argon (95%) and H_2_ (5%).

Three different laser scribing scan speeds have been used: 5.9, 2.7 and 1.2 mm/s. Two different materials coverage, as can be inferred from above: 400 and 850 µg/cm^2^. Finally, a single and a double laser pass were investigated.

From each sample, 20 Raman spectra were collected and statistically analysed. From the fitting process we calculated the ID/IG value for each spectrum, and reported such values as cumulative distributions. According to textbook definitions, the value of the cumulative distribution at I_D_/I_G_ = x is the number of occurrences in which I_D_/I_G_ < x. This has been calculated by the origin software for each samples using a column of I_D_/I_G_ as input values.

## Ethics Statement

Not applicable.

## CRediT Author Statement

**Vittorio Scardaci:** Conceptualization, Methodology, Data Curation, Investigation, Writing – Original draft preparation; **Giuseppe Compagnini:** Supervision, Writing – reviewing & editing.

## Declaration of Competing Interest

The authors declare that they have no known competing financial interests or personal relationships which have or could be perceived to have influenced the work reported in this article.
